# Prevalence of *Mycobacterium avium* Subsp. *paratuberculosis* in Feral Pigeons (*Columba livia*) Associated with Difficulties Controlling Paratuberculosis in a Bovine Herd (Fighting Bull Breed)

**DOI:** 10.3390/ani12233314

**Published:** 2022-11-27

**Authors:** Juan Seva, J. Manuel Sanes, Alberto Mas, Guillermo Ramis, Joaquín Sánchez, Ester Párraga-Ros

**Affiliations:** 1 Department of Anatomy and Comparative Pathological Anatomy, Faculty of Veterinary Sciences, Regional Campus of International Excellence “Campus Mare Nostrum”, University of Murcia, 30100 Murcia, Spain; 2 Department of Animal Production, Faculty of Veterinary Sciences, Regional Campus of International Excellence “Campus Mare Nostrum”, University of Murcia, 30100 Murcia, Spain

**Keywords:** paratuberculosis, fighting bull, cattle, pigeon, *Mycobacterium avium* subsp. *avium*

## Abstract

**Simple Summary:**

This study reports the 10 years (2011–2020) follow-up of a cattle herd, with a high prevalence of paratuberculosis (PTB) cohabiting with a population of pigeons. After 6 years without tests, the herd returned to a high prevalence, and the PTB program control was revised. Likewise, the presence of MAP in feral pigeons was studied to evaluate their role as a transmitter and/or reservoir, as well as their influence on controlling the PTB. Antibodies values according to the optical density (OD) determined by ELISA increased each year and could be used to isolate herds close to the cutoff point to improve PTB control. The maintenance of ELISA tests, even though there are no positive animals, is essential for the success of the PTB control. PCR results positive against MAP samples of intestine, foot skin, and feces were observed in pigeons. The follow-up carried out in this study made it possible to establish the wild pigeons as mechanical vectors of PTB in cattle after confirmation of MAP in the foot, as well as the possibility of a possible in intestine and feces. Although future studies should be carried out to clarify the true importance of these findings in the PTB control program in cattle, the control of pigeons in farm could be important.

**Abstract:**

A bovine herd with a high prevalence of paratuberculosis (PTB) cohabiting with a population of pigeons was studied (2011–2020). After finding the disease in 2011, annual monitoring was performed in 2012–2014 by obtaining blood samples for ELISA and intradermal tuberculinization (IT) tests for *Mycobacterium avium* subsp. *paratuberculosis* (MAP). Positive animals were eliminated. PTB prevalence dropped from 10% to 0% but returned to similar values (9.5%) after 6 years without tests. In all animals, Ac values according to the optical density (OD) determined by ELISA increased each year and could be used to isolate herds close to the cutoff point to improve PTB control. Possible reservoirs were considered after evaluating the little success of the PTB control program, and the population of feral pigeons was studied. Specifically, 10% of the pigeon population (*n* = 13) was necropsied. Samples of intestine, feces, and foot skin for PCR study for MAP and samples of terminal intestine for histopathological analysis were taken. Eleven pigeons were PCR-positive against MAP, in the intestine (10/11), foot skin (3/11), and feces (1/11). The presence of MAP in pigeon feet could demonstrate its role as a mechanical disseminator of PTB, while the presence in pigeon intestine and feces could also suggest its role as a reservoir.

## 1. Introduction

Paratuberculosis or Johne’s disease (PTB) is a worldwide animal health problem mainly in beef and dairy herds. It is chronic and debilitating enteritis caused by *Mycobacterium avium* subsp. *paratuberculosis* (MAP) [1]. The disease can have serious production-limiting consequences that cause significant relevant economic loss in the herd due to reduced milk yield, low productivity, and increased culling of sick animals. The diagnosis of PTB is difficult due to long incubation times and the presence of subclinical animals, which can excrete MAP for very long periods. This fact is essential to control the disease since these animals are a source of infection for other members of the herd by maintaining contagion [1,2].

The tests recommended for the control and eradication of PTB are different depending on the disease and the objectives that are sought. The tests used by most countries for individual animal diagnosis are serum ELISA, individual fecal PCR or culture, and pathology. However, if the entire herd is considered, diagnosis is achieved by serum ELISA, pooled fecal PCR, and milk ELISA [3]. The combined use of different diagnostic tests, such as ELISA and real-time PCR tests, helps to identify and remove “shedders” earlier, in addition to reducing the hazard of infection for other healthy animals [4,5]. Antibodies to MAP antigens develop before and during the onset of clinical disease, where ELISA sensitivity is very high as the disease progresses [6]. Likewise, sensitivity is almost 100% by PCR from fecal cultures in advanced phases [5]. On the other hand, the intradermal tuberculin test (IT) is the first test that is usually performed since its easy implementation in large herds allows the early detection of those infected. Subsequently, confirmation and screening by serological or milk-based testing is required [7,8]. Usually, the specificity of FC is almost 100%, if the isolates obtained at fecal culture are confirmed to be MAP by molecular methods [5]. Although various nonmandatory MAP control programs have proven to be effective in reducing the prevalence of MAP in the herd [6], its eradication is difficult and can only be achieved in some cases [3,9].

Even though PTB was long considered only a ruminant disease, PTB has also been diagnosed in non-ruminant wild species such as lagomorphs, canids, mustelids, corvids, and murines [10,11,12]. Therefore, the existence of wild animals with PTB has been considered key for the spread of the disease due to their role as a reservoir [3]. Domestic ruminants reared in free-range conditions may be in contact with wildlife and their excreta when grazing, which causes inter-species transmission [11]. In Europe, the wild rabbit (*Oryctolagus cuniculus*) is possibly the largest wildlife reservoir of PTB, and infected populations may contribute to the persistence of infection in rural farms [12].

The role of feral and domestic pigeons as a vector to transmitter of diseases has been demonstrated [13]. Specifically, avian mycobacteriosis caused by *M. avium* subsp. *avium* affects all kinds of birds. However, more than 10 other species of mycobacteria have been known to infect birds, including MAP [14,15,16]. In wood pigeons, observed mycobacteria have been considered atypical strains of *M. avium* [17]. Thus, it was possible to isolate *M. avium* from wood pigeons responsible for producing clinical PTB in calves [18]. Free-ranging bird hosts of MAP could play a relevant role in the PTB epidemiology of domestic ruminant cattle [14]. However, MAP has not been isolated in wild or domestic pigeons related to natural infections to date. In the same way, molecular typing that indicates transmission between domestic pigeons and cattle has not been previously performed, as it was between rabbits and cattle [10,19].

The aim of this study was to evaluate a PTB control program in a cattle herd that, after 6 years without PTB, returned to a high prevalence. Likewise, the aim was to confirm the presence of MAP in feral pigeons in this herd and evaluate their role as a transmitter and/or reservoir of the agent, making it difficult to control the PTB disease.

## 2. Materials and Methods

### 2.1. Cattle

**Figure 1:**
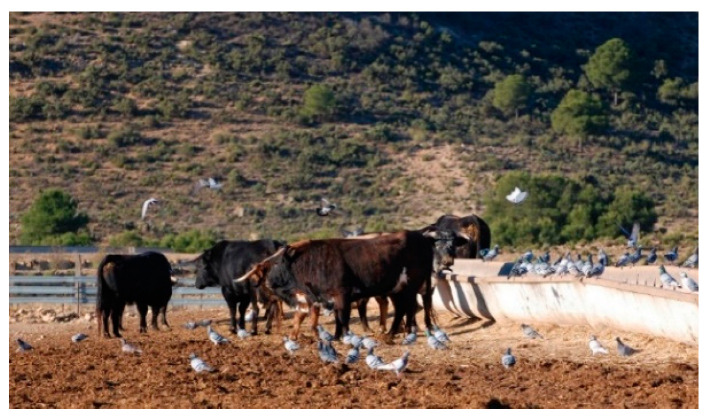
Fighting bulls sharing habitat with pigeons. Pigeons step and peck in the stool areas of the bulls and perch on the feeders.

The study was carried out in a herd of a fighting bull breed between 2012 and 2020 located in the southeast of Spain. The farm had an extensive breeding system and was following the mandatory program for the National Eradication of Bovine Tuberculosis (National Royal Decree 1939/2004, which regulates the health qualification of fighting bull bovine herds and the movement of animals belonging to them). The herd was divided into five lots of breeding animals, separated by fences of approximately 1 ha (Figure 1). In addition, another four separate lots of animals for sale of different ages were present. The animals remained together from mating until pup weaning at 8–10 months. The lots of breeding animals were made new each year with the incorporation of replacement animals from the farm itself or elimination of animals. The productive conditions on the farm are closed flow, without the incorporation of animals from outside. The farm has a perimeter fence that prevents the entry of other wild animals. The previous sanitary status in 2011 was characterized by the high prevalence of PTB (10%) as previously demonstrated [20].

### 2.2. Pigeons

The cattle farm has a loft with approximately 120 feral pigeons (*Columba livia*). It is in the immediate vicinity of the herding close contact with animals, drinkers, and feeders (Figure 1). A representative sample of the pigeon population (10%) was taken for the study (*n* = 13). The animals were captured by traps, stunned with anesthetic (isoflurane) inhalation, and euthanized by sectioning the jugular vein in accordance with the ethical standards of animal welfare. Subsequently, the necropsy was performed in the necropsy room of the Veterinary Faculty at the University. Samples of the terminal small intestine, feces, and foot skin were frozen for the molecular study. Moreover, other samples of the terminal small intestine in formol 10% were taken for the histopathological study.

### 2.3. Intradermal Tuberculinisation (IT) Test

IT tests were performed by public veterinary inspectors following the European Commission guidelines (EU Council Directive 64/432/CEE and National Royal Decree 1939/2004, which regulates health qualification of fighting bull breed herds and movement of animals) using PPD-avium and PPD-bovine (CZ Veterinaria, S.A., O Porriño, PO, Spain). Animals were injected intradermally with 0.1 mL (0.1 mg, 2500 CTU) of PPD-avium (D4 ER MAP strain) and 0.1 mL (0.1 mg, 2500 CTU) of PPD-bovine (AN-5) on the right side of the mid-cervical region. The injection site was visually inspected, and skin thickness was evaluated with calipers before and 72 h after inoculation. An animal was considered PTB-positive whenever there was an increase in thickness equal to or higher than 4 mm compared to the reaction point to injection, when signs such as diffuse edema, exudation, pain, or inflammatory reaction of the lymphatic ducts at the injection site were present, or when lymph nodes were obvious.

### 2.4. Serological (ELISA) Test

Serum of bovine blood samples was used for the detection of antibodies to MAP using a commercial indirect ELISA kit (PARACHEK^®^ 2, Thermo Fisher Scientific, Waltham, MA, USA) specifically for ruminant species. Reading was carried out at 450 nm using a plate reader, with 620 nm as reference wavelength at 30 min. Reference absorbance values as optical density (OD) were determined from the means of positive and negative controls according to the manufacturer’s instructions. Results were calculated after incubation. The cutoff point was calculated as the mean of two negative controls +0.150. Those whose sera presented absorbance values higher than cutoff point positive animals were considered.

### 2.5. Interpretation Criteria in the PTB Monitoring Program

The criterion used on tests was the following: ELISA- and/or IT-positive animals were considered PTB-positive. These positive animals were eliminated from the herd 1 week after blood sampling. The agreement between diagnostic techniques was established using the kappa index.

### 2.6. Histopathological Study

Samples fixed in 10% formalin were routinely processed in paraffin for histopathological evaluation. Two histological sections were obtained from each sample. One section was stained with hematoxylin and eosin and the other one with Ziehl–Neelsen (ZN) stain to evaluate the presence of acid-fast bacilli.

### 2.7. Molecular Study by Real-Time PCR

For DNA extraction, 20 mg of ileum and feces samples were taken and processed following the protocol for tissues of the commercial kit used (Gene JET Genomic DNA Purification Kit, Thermo Fisher Scientific). Foot skin samples were immersed in PBS for 24 h beforehand to obtain the remains adhered to them in suspension. That suspension (400 µL) was used with the same commercial protocol. DNA was quantified at 280 nm using a Nanodrop 1 (Thermo Fisher Scientific), and real-time PCR was performed in duplicate as described previously for the M. tuberculosis complex and M. avium complex [21]. Oligonucleotide primers myc1 (5′–GAGTAGGTCATGGCTCCTCC–3′) and myc3 (5′–CATGCACCGAATTAGAACGT–3′) were initially used to differentiate between M. tuberculosis complex and M. avium complex [22]. Positive samples for M. avium complex were tested to identify MAP using f57a (5′–GGTCGCGTCATTCAGAATC–3′) and f57b (5′–TCTCAGACAGTGGCAGGTG–3′) oligonucleotide primers, which specifically amplify a fragment of the gene f57 which is present in MAP DNA [22].

Real-time PCR was performed using a Fast Start Universal SYBR GREEN master mix (Roche) in 25 µL reaction volumes containing 5 µL of template and 10 pmol of each primer. To check the specificity of the amplified products, dissociation curve analysis was performed as previously described [21]. Negative controls (PCR analysis without DNA template) were used to detected possible contamination of MAP in the tissues, and bovine positive samples with MAP were used as positive controls. No contamination was detected in the samples.

## 3. Results

### 3.1. Monitoring of PTB in Cattle

**Figure 2:**
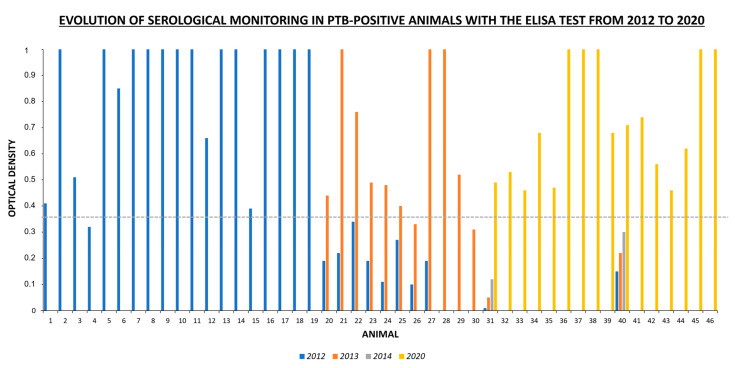
Optical density values with ELISA test in PTB-positive animals (*n* = 46) and their evolution during the studied period. The dashed line indicates the cutoff point established by the ELISA technique (0.36). The animals were considered positive when their serological indices were higher than the cutoff point. The animals whose serological indices were below the cutoff point were monitored in subsequent checkups and were eliminated once they were positive. After the free status of PTB reached in 2014, the serological control from 2015 to 2019 was not performed until the reappearance of the disease in 2020.

The high prevalence of PTB in the herd previously demonstrated in 2011 [20] and the beginning of subsequent sanitary, control, and monitoring actions resulted in a progressive decrease in prevalence in the following years. In 2012, the PTB prevalence dropped by 7.45% (Table 1), where 19 of 255 animals were considered PTB positive, 19/19 by ELISA and 8/19 by IT. Only an animal negative for serology was positive for IT, but it showed a specific Ac by OD against MAP just below the cutoff point. In 2013, 11 animals were considered PTB-positive, 11/11 by ELISA and 2/11 by IT. In this year, eight of them already had specific Ab values by OD to MAP, albeit below the cutoff point to consider them as positive in 2012. However, it was observed that these values in all animals increased from 1 year to another, becoming positive in 2013 (Figure 2). This year, the disease showed an important downward trend with a prevalence of 4.7% (Table 1), where the IT test was not sensitive enough to detect most of the positive animals since only 2/11 were positive with this technique. After analysis of the kappa index, the agreement observed between both diagnostic tests (IT and ELISA) was moderate (κ = 0.58) in 2012 (8/19) and poor (κ = 0.298) in 2013 (2/11). All animals were PTB-negative in the monitoring carried out in 2014 by ELISA and IT study.

However, 6 years later, some animals were observed with diarrheal symptoms that raised suspicions of the reappearance of PTB. ELISA tests indicated that 16 of the 172 animals sampled were positive, which supposes a prevalence of 9.3% (Table 1). In 2020, 105 of 172 animals were already in the farm since 2012 (61.04%), constituting two of the 16 new PTB positives detected (1.16%). In these two animals (animal 31 and 40 in Figure 2), levels of Ac were increased each year albeit below the cutoff point until 2020 when positives were considered. 

### 3.2. Confirmation of MAP in Pigeons

The necropsy and the histopathological analysis in pigeons did not reveal any macroscopic or microscopic lesions compatible with PTB. ZN-positive acid-fast bacilli were not observed in samples of intestine.

The positive samples showed two melting temperature peaks in PCR (87° and 89°) and an amplification of cycles 17–32 in most cases. Most pigeons (11/13) were PCR-positive against MAP. The detection of MAP was mainly identified in the intestine samples (10/11). In two of these animals, there was joint detection of MAP in intestine and foot skin, while there was joint detection in the intestine and feces in one animal. Only in one animal (1/11) was the presence of MAP exclusively on the foot skin (Table 2).

## 4. Discussion

This study shows the difficulties in the control of PTB in a cattle herd with an extensive rearing system and high prevalence of PTB, which coexists with confirmed MAP-positive feral pigeons. Nevertheless, it is not necessary to have a wildlife reservoir host to explain the pattern of disease development in these cattle. However, it is plausible that the feral pigeons were exposed, may have contacted and consumed bacteria, and may have tested positive in PCR to perpetuate PTB on this farm in conjunction with the inadequate herd testing regime, as it was insufficient over time.

A bovine herd with previous positivity for PTB was monitored by serological ELISA and IT for MAP for 4 years (2011–2014), and positive animals were eliminated. The use of ELISA tests as a PTB control strategy to reduce the prevalence of the disease has been widely verified [3,6]. In this study, it was thought that the disease had been eliminated in 2014 since all the animals tested were negative. For this reason, no new tests were carried out on the farm in subsequent years. However, 6 years later, new clinical cases of the disease appeared with twice the prevalence of that found in the last checkup. This fact reflects the difficulty to eliminate PTB, as already shown by other authors [3]. In this way, identification and elimination of clinically diseased and/or sub-clinically infected animals, i.e., “test and cull” [23], were not enough to eliminate PTB. The traditional ELISA test has several advantages as a good method to assess the prevalence of PTB [3,7]. Commercial ELISA has a good sensitivity (>70%) and specificity (99%) for the detection of this disease in cattle [24,25], and, although the sensitivity is very high during the advanced phases [6], it Is very low in the subclinical and silent stages [5,20]. On the contrary, IT has high sensitivity in the early stages of the disease [7]. Despite using both tests (ELISA and IT) that guarantee high sensitivities in the initial and final phases of the disease, in our case, sub-clinically animals infected by MAP were not detected in the 2014 checkup. The average life of the breeding cattle on this farm is long (some can live 20 years). Thus, in 2020, 105 of the 172 animals were already present in 2014 with negative results for the tests. Nevertheless, two of them were positive later; thus, any of them could have spread the infection in the farm if they had subclinical disease. In fact, 2/16 positives animals for PTB in 2020 were alive in 2014 but negative for IT and with OD values for ELISA below the cutoff point. In the same way, young animals (under 6 months) contaminated by vertical transmission through the placenta [26] could have been the trigger for the onset of the disease. These animals were not tested with ELISA and IT by the regulations applied on the farm. Despite the long incubation period for PTB [2] and the high number of animals older than 6 years in these herds, most new positive animals (14/16) were incorporated in the last 6 years. However, these long incubation periods may favor the reappearance of previously undetected PTB-positive animals that can excrete MAP and spread the disease mainly through fecal–oral contact [27]. In this sense, continuing to carry out ELISA tests for MAP for more years could have improved the results of our PTB control program. Likewise, testing replacement animals before incorporating them into the herd could have helped detect infected animals that were later positive for PTB.

The agreement between the two diagnostic tests (ELISA and IT) used to monitor the PTB was low and decreased from 2012 (κ = 0.518) to 2013 (κ = 0.298), where, of 11 ELISA-positive animals, only two were positive for IT. According to different authors [4,5], the changes in sensitivity observed for these tests depend on the stage of the disease in the animals since IT positives decrease as the disease progresses. The specificity for the ELISA test is close to 100% [18], with at least 30 animals positive for PTB on the farm. The low sensitivity of the IT test observed (10/30) could be related to the fact that a high number of animals were in advanced stages of the disease [7], when the ELISA test presents the highest sensitivity [18], similar to PCR of fecal culture [5]. This farm with a high prevalence of PTB showed a low sensitivity for IT. In 2011, according to necropsies and histopathological diagnosis, animals were in advanced stages of the disease (20). In this way, it is possible that, in farms with a low prevalence of PTB, IT presents greater sensitivity since the animals may be in earlier stages of the disease. We also found some animals with substantial IT reaction and low antibody values according to OD, with loss of sensitivity to the ELISA test, which could have been because they were in the early stages of the disease [7]. Nevertheless, sensitivity deficiencies to the ELISA test related to the antigens used in the tests have also been observed, in addition to the variation of sensitivity according to the country when the animals were infected by different strains [28].

In the same way that the level of antibodies detected with ELISA tests increased 2 years before the time of mycobacterial elimination [29], the present study clearly showed, in all positive animals, an increase in specific antibodies values against MAP by OD from 1 year to the next. In addition, the growth antibody level observed in two animals for 8 years could explain the long incubation period of the disease [2]. Although ELISA is not considered a quantitative test [7], these results can be very interesting to consider for improving PTB control programs. Thus, new herds could be created with levels of OD close to the cutoff point without removing animals, with special monitoring and without contact with negative herds, since they could be excretory and polluting at any time before the next checks. Thus, in case any animal becomes positive, it could be eliminated without compromising transmission in the rest of the herd.

PTB is not very prevalent in extensive breeding cattle since environmental exposures to mycobacteria are very low, as normally occurs in these bullfighting cattle, unlike intensive breeding [3]. However, the reappearance of a high PTB prevalence (9.3%) after eliminating the disease by tested monitoring led to the possibility of another external source of contagion among bovines studied. Due to the habitat of this cattle herd and the maintenance of PTB after an unsatisfactory control program, the wildlife animals were studied. In these cattle farms, the presence of wild pigeons is constant and in close contact with the bovines. After carrying out a representative study of the population, which constituted approximately 10% of the pigeon population, the positivity for MAP was found in a very high percentage of the animals studied (84.6%). MAP has been found in the intestine [14,16] of birds located near bovine farms positive to PTB. However, although mycobacteria of wood pigeons have been responsible for producing clinical PTB in calves [18], in feral pigeons, the presence of MAP has not been demonstrated until now. Therefore, pigeons must be considered as possible PTB transmitters or reservoirs.

The feeding system used for fighting bulls is in a grinding format with whole or fragmented cereal grains completely resistant to ruminal digestion. Thus, grains and or fragments of them could be appear in the feces since the continuity of the seed surface prevents microbial colonization and its decomposition [30]. For this reason, in our farm it was observed how the pigeons pecked and ingested food in the defecation areas of the cattle (Figure 2). In addition, this mycobacterium survives up to 55 weeks in unfavorable environments, which favors contamination [31]. In these actions, pigeons can impregnate the foot skin with mycobacteria and spread PTB by depositing in other locations such as fodder, feeders, or waterers of cattle. Thus, the detection of MAP by PCR in the skin samples from the pigeon feet in this study could demonstrate the role of mechanical vectors that birds may have for the spread of diseases [32]. In this sense, the control of pigeons in PTB control programs can already be justified. Similarly, pigeons can orally ingest certain amounts of MAP excreted when collecting the remains of cereals contaminated by feces. However, their role as a carrier of the disease is not clearly demonstrated despite the detection of MAP DNA in the intestine and feces of pigeons. The DNA can correspond to the remains of mycobacteria digested by the pigeons, and cultures for MAP would need to be performed to ensure that they were alive and played a role as a reservoir and carrier of PTB. The mycobacterial load in the intestine was probably low since no macroscopic lesions were evidenced, while mycobacteria were not observed by histopathology; however, it was detected due to the greater sensitivity of the PCR technique [20]. Therefore, it is possible that detection of MAP by PCR in the intestine and feces of pigeons could indicate the spread of the disease when they excrete on the grass or feeders, opening the possibility of transmission between species [10]. However, culture studies for MAP must be performed to determine that MAP is alive within the pigeons, and these can be considered a reservoir of PTB.

The environmental study determined pigeons as possible risk factors involved in the maintenance of the PTB in the farm, which could represent a mechanical vector and a possible reservoir [14]. These facts could indicate that control of the pigeon population in our farm would help in PTB eradication programs. In addition, despite the rural location, the closed-flow productive conditions and the perimeter fencing of the animals that prevents the entry of other wild animals allowed us to discard them as reservoirs [12,14]. However, MAP can circulate among wildlife hosts including deer species and rabbits, and available epidemiological data suggest that infection of cattle or sheep through contaminated pastures shared with wildlife is possible [12,14,33], but it is unlikely in this farm.

## 5. Conclusions

These findings could be relevant to improve PTB control programs since the reappearance of the disease was demonstrated despite the elimination of positive animals by the diagnostic tests (ELISA and IT). The special management and monitoring of animals that could be excretory of MAP (with OD levels close to the cutoff point), without eliminating them and without contact with negative herds, could improve PTB control. Periodic monitoring of the disease, even when the disease is no longer detected with the tests, seems necessary to avoid a subsequent increase in prevalence of disease.

The follow-up carried out in this study made it possible to establish the wild pigeons as mechanical vectors of PTB in cattle, as well as a possible reservoir, after confirmation of MAP in the intestine and feces. Therefore, control of the same in areas close to the farm could be important to help maintain a good health status. However, future studies should be carried out to clarify the true importance of these findings in the PTB control program in cattle.

## Figures and Tables

**Table 1:** Evolution of the PTB prevalence in breeding cattle. Annual diagnostic controls for PTB were carried out by ELISA and IT test from 2012 to 2014. In 2020, only ELISA tests were performed.

Year	Animals	Test	Animals PTB+	Prevalence PTB
2011 *	350 *	ELISA, IT *	35 *	10% *
2012	255	ELISA, IT	19	7.45%
2013	234	ELISA, IT	11	4.7%
2014	205	ELISA, IT	0	0%
2020	172	ELISA	16	9.3%

* Published data in Seva et al. (2014) [20].

**Table 2:** Positivity against MAP in PCR samples of intestine, feces, and foot skin of pigeons.

Animal	PCR Intestine	PCR Faeces	PCR Foot Skin
1	+	−	+
2	+	+	−
3	−	−	+
4	+	−	−
5	−	−	−
6	+	−	−
7	+	−	+
8	+	−	−
9	+	−	−
10	+	−	−
11	+	−	−
12	−	−	−
13	+	−	−
Total	10	1	3

## Data Availability

The data presented in this study are available on request from the corresponding author.
